# The RuvABC Holliday Junction Processing System Is Not Required for IS*26*-Mediated Targeted Conservative Cointegrate Formation

**DOI:** 10.1128/spectrum.01566-23

**Published:** 2023-06-26

**Authors:** Carol H. Pong, Jade E. Peace, Christopher J. Harmer, Ruth M. Hall

**Affiliations:** a School of Life and Environmental Sciences, The University of Sydney, New South Wales, Australia; McGill University

**Keywords:** IS*26*, IS*26* translocation, RuvABC

## Abstract

The insertion sequence IS*26* plays a key role in the spread of antibiotic resistance genes in Gram-negative bacteria. IS*26* and members of the IS*26* family are able to use two distinct mechanisms to form cointegrates made up of two DNA molecules linked via directly oriented copies of the IS. The well-known copy-in (formerly replicative) reaction occurs at very low frequency, and the more recently discovered targeted conservative reaction, which joins two molecules that already include an IS, is substantially more efficient. Experimental evidence has indicated that, in the targeted conservative mode, the action of Tnp26, the IS*26* transposase, is required only at one end. How the Holliday junction (HJ) intermediate generated by the Tnp26-catalyzed single-strand transfer is processed to form the cointegrate is not known. We recently proposed that branch migration and resolution via the RuvABC system may be needed to process the HJ; here, we have tested this hypothesis. In reactions between a wild-type and a mutant IS*26*, the presence of mismatched bases near one IS end impeded the use of that end. In addition, evidence of gene conversion, potentially consistent with branch migration, was detected in some of the cointegrates formed. However, the targeted conservative reaction occurred in strains that lacked the *recG*, *ruvA*, or *ruvC* genes. As the RuvC HJ resolvase is not required for targeted conservative cointegrate formation, the HJ intermediate formed by the action of Tnp26 must be resolved by an alternate route.

**IMPORTANCE** In Gram-negative bacteria, the contribution of IS*26* to the spread of antibiotic resistance and other genes that provide cells with an advantage under specific conditions far exceeds that of any other known insertion sequence. This is likely due to the unique mechanistic features of IS*26* action, particularly its propensity to cause deletions of adjacent DNA segments and the ability of IS*26* to use two distinct reaction modes for cointegrate formation. The high frequency of the unique targeted conservative reaction mode that occurs when both participating molecules include an IS*26* is also key. Insights into the detailed mechanism of this reaction will help to shed light on how IS*26* contributes to the diversification of the bacterial and plasmid genomes it is found in. These insights will apply more broadly to other members of the IS*26* family found in Gram-positive as well as Gram-negative pathogens.

## INTRODUCTION

IS*26* is one of the most important insertion sequences (IS) mobilizing antibiotic resistance genes and plays a role in recruiting antibiotic resistance genes into the mobile gene pool. IS*26* also shapes the large clusters of antibiotic resistance genes found in multiply and extensively resistant Gram-negative pathogens by bringing in further resistance genes or removing others. Though its activity was initially characterized in the 1980s (1, 2), why IS*26* was so much more influential than the multitude of other IS that were identified (3) had remained unexplained.

IS*26* appears to be a classical IS that is bounded by terminal inverted repeats (TIR), encodes a DDE transposase, and generates a target site duplication (TSD; of 8 bp). However, early work demonstrated that IS*26* differed from other classical (DDE) ISs in that, rather than moving to a new location as a discrete entity, when the target is in a different DNA molecule it forms a cointegrate made up of the donor and target molecules linked by directly oriented copies of the IS (1, 4, 5). IS*26* can also generate an inversion or a deletion of adjacent DNA when the target is in the same molecule. This reaction mode was originally called “replicative” transposition but is now known as “copy-in” cointegrate formation, inversion, or deletion (5, 6). That the copy-in route exclusively forms cointegrates when a second DNA molecule that includes no copies of IS*26* is the target was recently confirmed using a more sensitive assay (4). Homologous recombination is needed to reseparate the two molecules in a cointegrate product and complete the transposition of a single IS*26* copy now surrounded by a duplication of the 8-bp target site (7). Formation of a cointegrate as the end product has consequences for the structure and movement of the pseudo compound transposons that are bounded by copies of IS*26*, as described in some detail elsewhere (7). These features contrast with ISs that use the better-studied copy-out/paste-in or cut-out/paste-in mechanisms, where the complete transposition process is undertaken by the IS-encoded transposase (3, 6, 8, 9).

**FIG 1:**
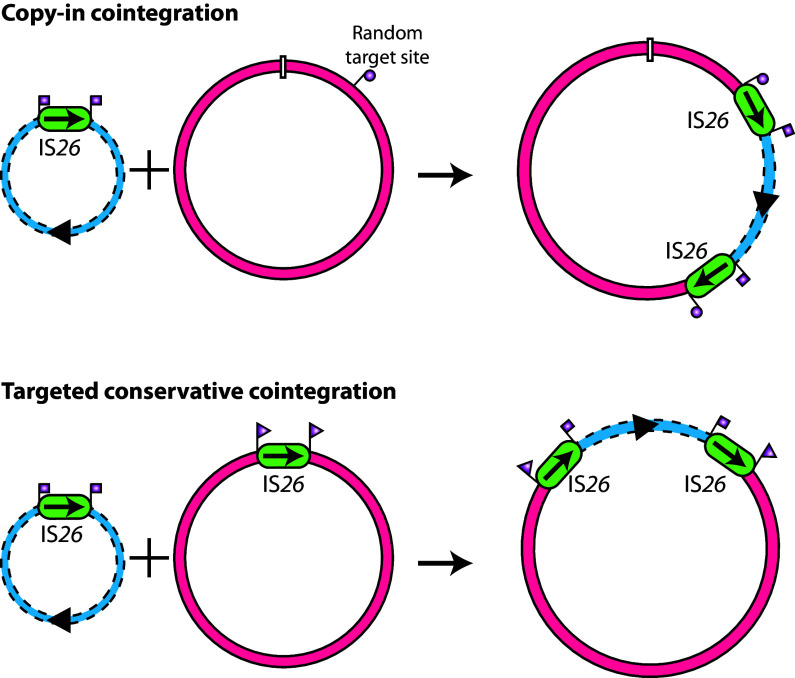
The two routes to cointegrate formation: untargeted copy-in cointegration and targeted conservative cointegration. IS*26*s are shown as rounded boxes with arrows indicating the extent and orientation of the *tnp26* gene. The two replicons, alone or in the cointegrate, are indicated by red or blue lines. The flanking 8-bp target site duplications are indicated. To facilitate tracking, the original IS*26* copies are shown with surrounding 8-bp TSDs, denoted by square or triangular flags. The newly formed TSD in the copy-in reaction is denoted by circular flags.

Further insight into the unique properties of IS*26* was published in 2014 when evidence for a previously unidentified mode of cointegrate formation also undertaken by IS*26* was reported (5). This reaction mode requires that both DNA molecules participating in the reaction that forms the cointegrate contain a copy of IS*26* (Fig. 1). Hence, it could not be detected by the assays used in early studies. In this mode, which occurs at a substantially higher frequency than copy-in cointegrate formation, the reaction is targeted in that the reaction clearly has occurred between the two copies of IS*26*. Examination of the products revealed that this reaction mode was conservative in that no new copies of IS*26* were generated and no bases were lost or gained in the surrounds (5). These features resemble site-specific recombination rather than transposition using a DDE transposase. As a consequence, the cointegrate product resembles that formed by homologous recombination occurring within the boundaries of the two IS*26*s. However, the reaction was IS*26*-mediated, as it occurred in a recombination-deficient strain and required the IS*26* transposase Tnp26 (5). Targeted conservative cointegrate formation also occurs at a higher frequency than homologous recombination between two inactivated copies of IS*26* in a recombination-proficient background (10). More recently, a number of ISs that are related to IS*26*, namely, IS*1216* and IS*25*7 (IS*431*), that are found in Gram-positive bacteria, and IS*1006* and IS*1008*, found predominantly in Acinetobacter species, have been shown to perform the same targeted conservative reaction (11, 12). Hence, the targeted conservative reaction appears to be an ability of IS in the recently defined IS*26* family (13).

A comparison of the transposases encoded by the IS in the IS*26* family has provided some insight (13), as it revealed an unusual conserved motif in a short segment at the N terminus in addition to a trihelical helix-turn-helix (HTH) domain, which is the DNA binding domain (14), and the catalytic domain. The N-terminal segment includes two planar aromatic residues, usually F (phenylalanine) but occasionally Y (tyrosine), separated by 4 amino acids (aa). The F4F motif has not been detected in ISs from any other IS family and is likely key to the unusual capabilities of IS*26* family members. More recent *in vivo* and *in vitro* studies have demonstrated a role for both of the N-terminal features of Tnp26, namely, F4F and HTH, in activity and in binding of Tnp26 to the IS*26* TIR (14).

To date, studies of the detailed properties of the targeted conservative mechanism are limited. By changing the outer two bases of the TIR at one or both ends of one of the participating ISs, which should block strand transfer at the affected end, it was shown that strand transfer at only one end was needed and that either the left or the right ends could be paired productively (15). Recently, we extended these findings by examining the properties of IS*1006*/*1008*, a naturally occurring hybrid of the closely related IS*1006* and IS*1008*, that encodes a transposase identical to Tnp1008 (12). IS*1006*/*1008* in one plasmid was able to form cointegrates with a second plasmid carrying either the same IS or IS*1008* at similar frequencies via the targeted conservative cointegration reaction (12). However, all cointegrates in the IS*1006*/*1008* reaction with IS*1008* formed at the end where the DNA sequences are identical, and it was concluded that sequence identity allowing branch migration was needed for a productive conservative reaction. In addition, DNA sequence identity was not the only requirement, as IS*1006*/*1008* was unable to form cointegrates via the targeted conservative route when partnered with IS*1006*, despite 175-bp DNA sequence identity at one end, and it was concluded that the modest number of amino acid differences between Tnp1008 and Tnp1006 (see Fig. 1 in reference 12) precluded a productive interaction. To explain these findings, we proposed a model in which a nicked Holliday Junction (HJ) formed by Tnp26 must migrate toward the other IS end but does not need to reach it (12). Progression of the HJ could be undertaken by RuvAB or RecG or both, and the HJ could resolved by RuvC. Alternatively, replication could be involved.

Here, we have discovered evidence for branch migration in the products of crosses involving a wild-type IS*26* and a mutant. The effects of deletion of the *recG* or *ruvA* genes, which are involved in branch migration, and of *ruvC*, which is required for HJ resolution, on the ability of IS*26* to complete the Tnp26-initiated targeted conservative reaction were also examined.

## RESULTS

### Mutations near one of the IS ends impede the reaction at that end.

**FIG 2:**
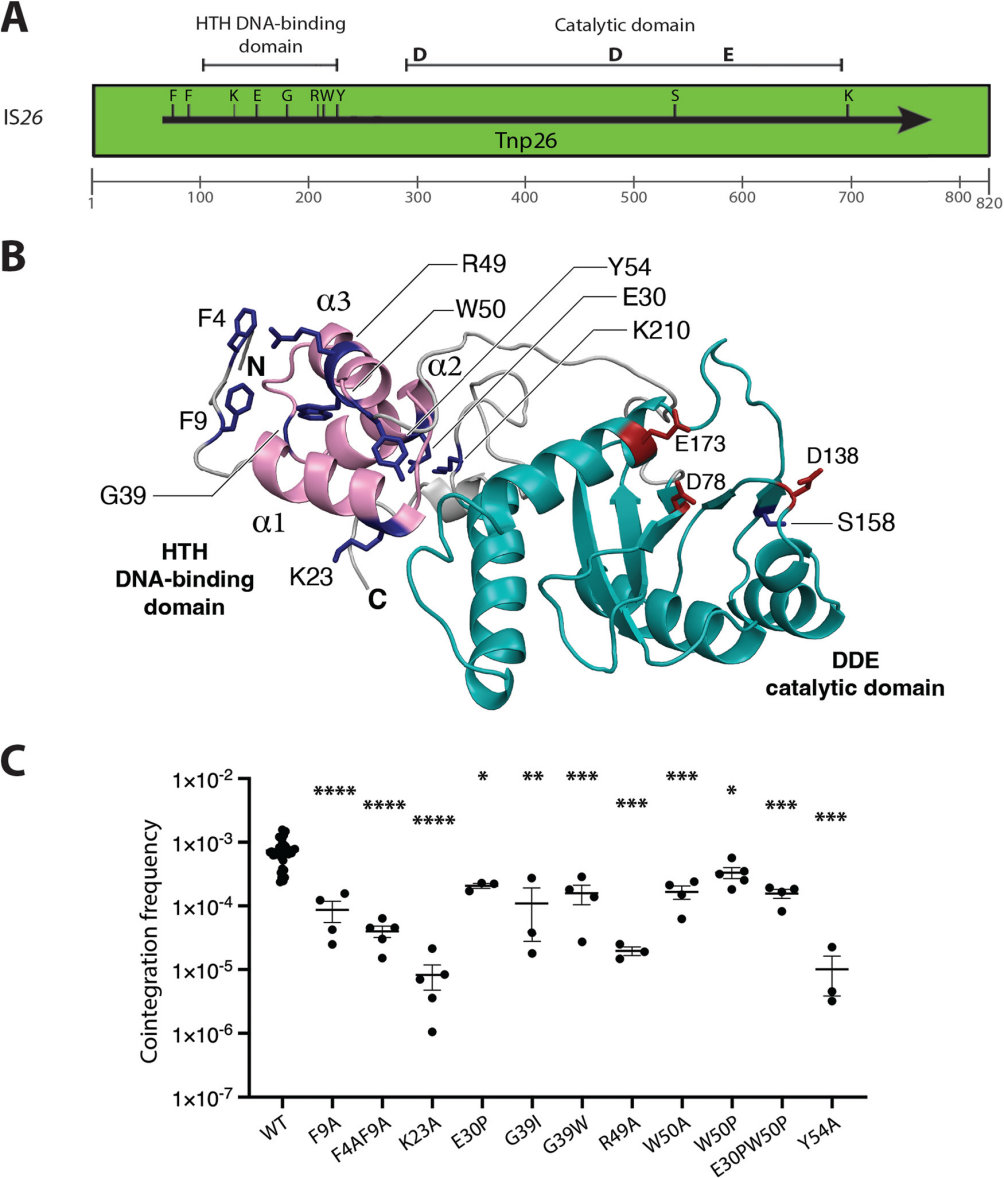
(A) Schematic representation of IS*26*. The extent and orientation of the *tnp26* gene are indicated by a black arrow. Amino acids affected by the substitutions are marked by the black letters. The Tnp26 HTH DNA-binding domain and the DDE catalytic domain are marked at the top. A scale bar for nucleotide distance from the left end is shown below. The positions of the DDE catalytic triad are marked by bold letters. (B) Tnp26 model generated using Phyre2 with the HTH DNA-binding domain and DDE catalytic domain colored pink and light blue, respectively. The DDE catalytic residues (D78, D138, and E173) are colored red and labeled. The residues targeted for substitution are colored dark blue and labeled. (C) Frequency of cointegration between pRMH977 mutant derivatives with R388::IS*26* (*n *> 3). Significance, in comparison to WT, was determined using a one-way ANOVA and Šidák multiple-comparison test.

**FIG 3:**
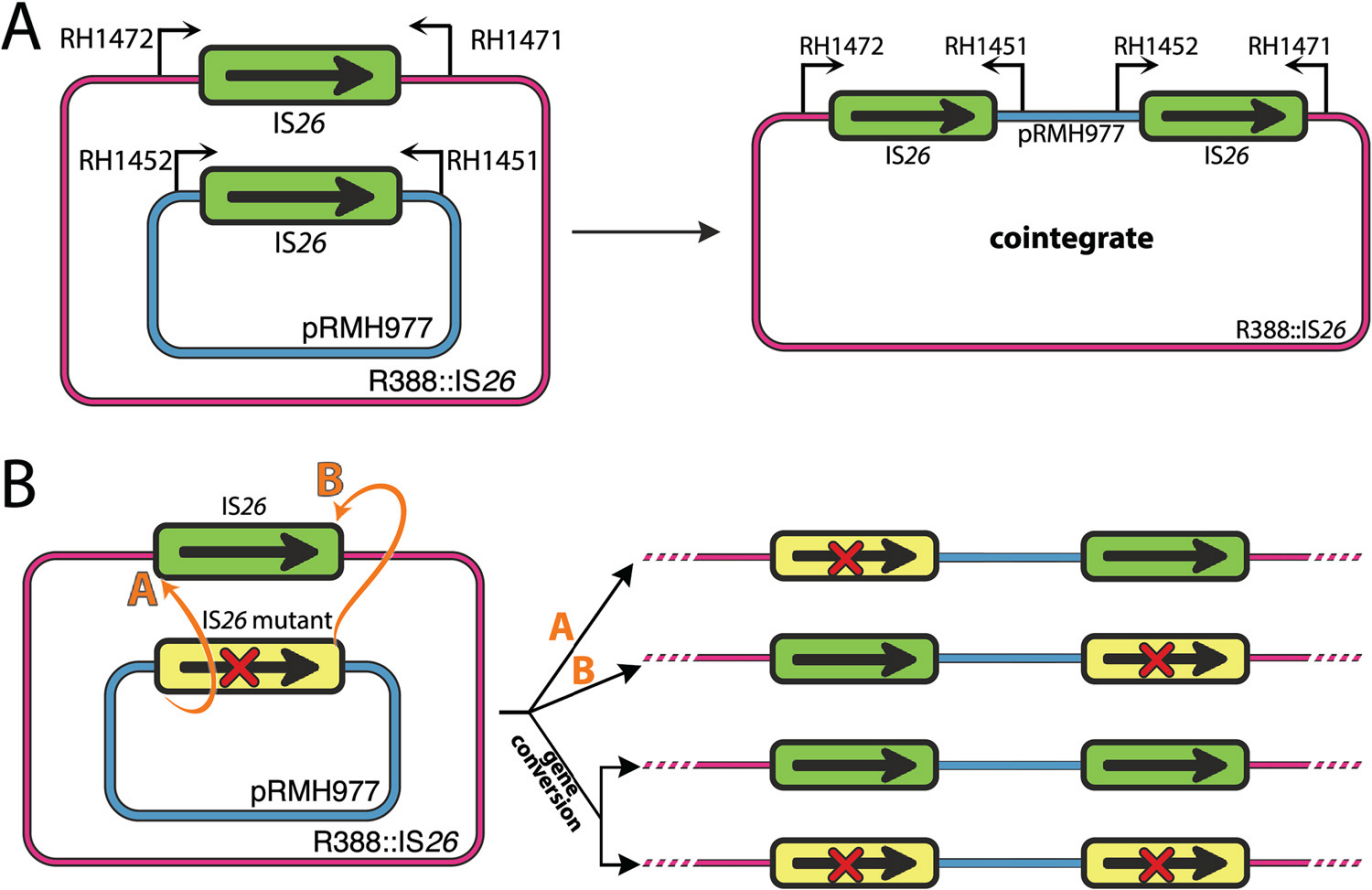
Analysis of IS positions in cointegrates. (A) Schematic of the reacting plasmids and the cointegrate product formed by reaction between the left or right IS ends, showing locations of the primers used to amplify the IS*26* at each junction in cointegrates. The primer sequences have been reported elsewhere (5). (B) Determination of the location of the mutant and wild-type ISs. Sequencing of PCR products amplified using primers shown in panel A indicate the IS end used.

We recently reported the construction of a series of IS*26* derivatives in which alterations to the DNA sequence near the left end of the IS had been introduced in order to alter specific amino acid residues in either the N-terminal F4F motif or the HTH domain (14). The positions of these mutations are shown in Fig. 2A and the locations of the amino acids in Tnp26 that they affect are shown in Fig. 2B. The mutations generally reduced the efficiency of the copy-in reaction to levels below the limit of detection (14). For the targeted conservative reaction, when both participating ISs included the mutation, the efficiency was also substantially reduced (at least 2 orders of magnitude) (14). Here, the impact of these nucleotide substitutions (generally two adjacent base pairs) and consequent single amino acid alterations on crosses between each mutant IS*26* and the wild type-IS*26* were investigated. In this configuration, only more modest reductions (about 10-fold) in the frequency of cointegrate formation were observed (Fig. 2C). In all cases, the reaction was targeted and conservative, as evidenced by amplification of the junctions (for three cointegrates from each of three independent crosses) that would be produced if the reaction were conservative (see Fig. 3A for methodology). The relatively small effect of introducing one mutant that is inactive on its own into the reaction likely reflects an *in* *trans* action of the Tnp26, which usually acts *in* *cis*.

Initially, the two ISs in a number of cointegrates were sequenced to enable construction of an R388::IS*26* derivative that included the mutation. In the course of that work, it was noticed that the mutation appeared significantly less frequently than predicted (if reactions at the two ends were equally efficient) in the IS*26* on the left, as shown in Fig. 3, and occasionally cases of gene conversion had occurred. To determine if the presence of the mutations near the left IS end had indeed reduced the probability that the reaction occurred at that end, for several of these crosses the two ISs in a number of further cointegrates from multiple independent reactions were both amplified and sequenced. Generally, the original base substitutions were present in only one of the two ISs in the cointegrate. However, the location in the cointegrate of the IS containing the mutations (left or right in Fig. 3B), which is indicative of which end of the two ISs participated in the strand transfer reaction (Fig. 3B), revealed a significant bias in the distribution of products toward those formed by a reaction at the right end of the IS (Table 1). This effect was seen for 2-bp differences located even over 200 bp from the outer end. As the reaction occurs at the opposite end to that carrying the mutation, we concluded that even just two adjacent nucleotide substitutions near one end (88 to 244 nucleotides away) is sufficient to strongly affect the efficiency of completion of a reaction at that end after an initial strand exchange carried out by Tnp26.

**TABLE 1:** Location of the IS*26* mutation in cointegrates formed between mutated pRMH977 derivatives and R388::IS*26*

Tnp26 (encoded on pRMH977)				No. of cointegrates screened (no. of independent assays)	Location in cointegrate of IS*26* mutation			
Codon change^*a*^	nt positions^*b*^	aa substitution(s)	No. of bases changed		Left	Right	Both	Neither
On left in IS								
TTT>GCT	88–89	F9A	2	12 (4)	2	10	0	0
TTC>GCC (4), TTT>GCT (9)	77–78, 88–89	F4A, F9A	4	15 (5)	0	14	1^*c*^	0
AAA>GCA	130–131	K23A	2	17 (4)	1	14	0	2
GAG>CCG	151–152	E30P	2	13 (3)	1	7	4	1
GAG>CCG (30), TGG>CCG (50)	151–152, 211–212	E30P, W50P	4	9 (2)	1	2	6	0
GGA>ATA	178–179	G39I	2	9 (3)	3	5	0	1
GGA>TGG	178, 180	G39W	2	8 (2)	1	6	0	1
CGC>GCC	208–209	R49A	2	10 (3)	4	5	1	1
TGG>GCG	211–212	W50A	2	9 (3)	2	5	2	0
TGG>CCG	211–212	W50P^*c*^	2	8 (3)	0	4	4	0
TAT>GCT	223–224	Y54A	2	9 (3)	1	8	0	0
On right in the IS								
TCT>TCA	535–536	S158^*d*^	1	15 (3)	7	0	1	7
AAA>GCA	691–692	K210A	2	15 (5)	15	0	0	0

a The changed nucleotides are underlined.

b Position of bare pair change relative to IS*26* (820 bp).

c Mutation encoding Tnp26 F9A was in the left IS*26* copy, and Tnp26 F4A was on the right.

d Silent mutation was introduced into Tnp26 S158.

To confirm this finding, we examined the effect of a substitution near the right end of the IS. The K residue at position 210 was replaced with an A by changing two bases in the codon, which is located 128 to 130 bp from the right end of the IS. In this case, the amino acid substitution did not significantly affect the efficiency of cointegrate formation (average, 5.1 × 10^−4^ from 5 determinations), but in all 15 of the cointegrates examined the reaction had occurred at the opposite end (Table 1). Likewise, a single base substitution in the codon for S158 located 285 bp from the right end, which did not alter the amino acid or affect the cointegration frequency (average, 6.5 × 10^−4^ from 3 determinations), yielded no cointegrates formed using the right end. Hence, it appears that the presence of just one or two nucleotide substitutions even over 200 bp from one end impedes the completion of a potential cointegration event initiated by a strand transfer at that end, and this is consistent with a requirement for branch migration in order to complete the reaction initiated by Tnp26.

### Gene conversion.

Gene conversion was also observed. In several cases, the mutation was not present in either IS copy in the cointegrate, and in other cases, the mutation was present in both (Table 1). These findings suggested that a HJ formed by the Tnp26 transposase at the outer end near the mutation can progress toward the other IS end, creating heteroduplexes, although progression past the mismatch would be much less efficient than when the two IS*26*s are the same. The mismatches generated could then be repaired by an undirected mismatch repair pathway.

### Effect of RecG and RuvA.

In E. coli, both RecG and RuvAB can facilitate branch migration (16). A derivative of AB1157 in which the *recG* gene was replaced by a kanamycin resistance gene was used to host the two plasmids containing IS*26*s involved in cointegrate formation. The reaction between two wild-type copies of IS*26* was not reduced in this background relative to the recombination-proficient parent AB1157 (Table 2). In both cases, the cointegration frequency was in the same range as previously reported for the reaction carried out in a recombination-deficient background (5, 11, 12).

**TABLE 2:** Effects of RecG, RuvA, and RuvC

Strain	Deletion	No. of replicates	Cointegration frequency	
			Range	Mean (SD)
AB1157	—	3	2.83 × 10^−4^–6.21 × 10^−4^	4.04 × 10^−4^ (1.88 × 10^−4^)
AB1157-*recG*	*recG*	3	2.91 × 10^−4^–1.52 × 10^−3^	1.44 × 10^−3^ (1.11 × 10^−3^)
JW1850	*ruvA*	5	2.52 × 10^−4^–7.44 × 10^−4^	1.83 × 10^−4^ (4.38 × 10^−4^)
JW1852	*ruvC*	3	1.02 × 10^−4^–9.91 × 10^−4^	4.52 × 10^−4^ (4.74 × 10^−4^)

Targeted conservative cointegrate formation also occurred at a normal frequency in strain JW1850, from the Keio Collection, which lacks the *ruvA* gene (Table 2). Hence, neither RecG nor RuvA is essential.

### Effect of RuvC.

Resolution of the nicked HJ formed at the reacting end could occur via replication through the structure or could involve the RuvC HJ resolvase. When the conservative reaction between two wild-type copies of IS*26* was measured in strain JW1852, which lacks the *ruvC* gene, the frequency of cointegrate formation was also similar to the frequencies reported for AB1157 (Table 2).

We considered the possibility that RuvC may be involved when the HJ cannot progress to the other end of the IS. To examine this possibility, the reaction of IS*1006/1008* with IS*1008*, which occurs exclusively via strand transfer at a single end (12), was examined in the RuvC-deficient strain. Again, the frequency of the targeted conservative reaction (average 6.9 × 10^5^; 3 determinations) was similar to that reported previously (12) for the same reaction in a recombination-deficient background (average, 5.6 × 10^5^). Hence, RuvC is not required.

## DISCUSSION

Little is known about the steps involved in processing the nicked HJ presumed to be formed by Tnp26 and other transposases in the IS*26* family. Here, a strong bias was observed toward the production of targeted conservative reaction products arising via strand exchange at the end distant from any nucleotide differences between the IS*26* copies involved in the reaction. This suggested that IS*26*-mediated strand exchanges occurring at the other end are either not processed or inefficiently processed to completion. However, when they are processed, gene conversion can occur, consistent with a branch migration step.

The results presented here eliminate one possible route to resolution of the HJ intermediate formed by the action of Tnp26 at one of the pairs of like IS ends of the two IS*26*s involved in the reaction, as RuvC, which is required for resolution of HJs, does not appear to be needed for resolution of this HJ. We obtained circumstantial evidence for branch migration in the form of the gene conversion observed in crosses between a mutant and a wild-type IS*26*, and this would presumably arise by undirected mismatch repair of a heteroduplex formed by branch migration. Elimination of RecG or RuvA, both of which can progress the crossover in a branched form, did not affect the efficiency of cointegrate formation. However, granted the functional redundancy of RecG and RuvAB, it cannot be concluded that these pathways are not involved, and construction of a mutant strain that lacks both pathways will be needed to investigate this further. However, as RuvC is not involved, the precise steps involved after the Tnp26 acts initially remain to be determined.

It has been proposed that, in the targeted conservative mode, the HJ formed by Tnp26 action at one IS end progresses to the other end of the IS, where it is resolved by a second strand transfer catalyzed by Tnp26 (17). However, this proposed route is not consistent with our previous finding that targeted conservative cointegrate formation continues to occur when the outermost 2 bp at one end have been altered (15), precluding involvement of that end in strand transfer. Our further observation that the reaction only occurs at one end in other cases where progression of an HJ would be possible only for part of the IS length at each end (12) is also inconsistent with this proposed route.

One possible scenario for processes leading to formation of a viable cointegrate is that branch migration would lead the crossover formed by Tnp26 away from the ends of the IS, leaving a nick at the outer end of one IS. If the nick has been ligated, this potentially opens the door to a second strand exchange event between the same pair of ends. This could lead to either effective reversal of the initial crossover or to a crossover involving the second pair of strands, effectively completing the reaction. Replication is also a potential alternate route to conversion of the HJ formed in the targeted conservative route into the final cointegrate product. Indeed, we recently proposed repeated replication through an incoming IS*26*-containing translocatable unit (also known as a TU) as a likely route to extensive gene amplification in both the copy-in and targeted conservative modes (18). However, if in the targeted conservative mode Tnp26 generates only a single-strand transfer (see Fig. 5 in reference 12), the 3′-end generated would prime replication away from the ISs. Hence, further work will be needed to examine the possibility that completion of the conservative reaction involves replication.

The key to understanding the targeted conservative mode may lie in a better understanding of the role of the characteristic F4F motif at the N terminus of Tnp26 and all transposases encoded by members of the IS*26* family. We have previously shown that replacement of either one of the F residues impacts activity and abolishes binding of Tnp26 to an IS end, consistent with a role in end recognition and binding. However, experimental evidence, derived from examining the effect of replacing different pairs of F residues in the Tnp26 produced by the reacting IS*26*, is consistent with a direct interaction between corresponding F residues in each Tnp26, and this is consistent with a role in dimer formation or stability (14). However, whether properties conferred by this motif can indeed explain the unusual abilities of ISs in the IS*26* family remains to be established.

## MATERIALS AND METHODS

### Strains and plasmids.

E. coli strains used in mating-out assays are listed in Table 3. Plasmids are listed in Table 4. Derivatives of pRMH977 (IS*26* cloned into pUC19; Ap^R^) (5) encoding amino acid substitutions in Tnp26 were constructed using site-directed mutagenesis, as previously described (5). Bacterial strains were routinely cultured with aeration in LB (Luria-Bertani; 0.5% (wt/vol) yeast extract, 1% (wt/vol) NaCl, 1% (wt/vol) tryptone) liquid or solid (LB broth, 1.5% [wt/vol] agarose) media at 37°C. Media were supplemented as appropriate with antibiotics (Sigma-Aldrich) at the following concentrations: ampicillin (Ap) at 100 μg/mL, nalidixic acid (Nx) at 25 μg/mL, streptomycin (Sm) at 25 μg/mL, and trimethoprim (Tp) at 20 μg/mL. Mueller-Hinton broth 2 (Becton, Dickinson) or Mueller-Hinton agar (Oxoid) were used when supplementing with Tp.

**TABLE 3:** E. coli strains used in the study

Strain name	Full genotype	*rec*/*ruv* allele^*a*^	Resistance phenotype	Reference
UB1637	*his lys trp strA rpsL*(Sm^R^)	Novel *recA*	Sm^R^	20
UB5201	*pro met gyrA*(Nx^R^)	Novel *recA*	Nx^R^	20
AB1157	F^−^ *thr-1 araC14 leuB6* Δ(*gpt-pro*)*62* l*acY1 tsx-33 qsr′-0 glnX44 galK2 rac-0 hisG4 rfbC1 mgl-51 rpoS396 rpsL31*(Sm) *kdgK51 xylA5 mtl-1 argE3 thiE1*		Sm^R^	
AB1157Δ*recG*	F^−^ *thr-1 araC14 leuB6* Δ(*gpt-pro*)*62* l*acY1 tsx-33 qsr′-0 glnX44 galK2 rac-0 hisG4 rfbC1 mgl-51 rpoS396 rpsL31*(Sm) *kdgK51 xylA5 mtl-1 argE3 thiE1*	Δ*recG263*::Km	Sm^R^ Km^R^	21
JW1850	F^−^ Δ(*araD*-*araB*)*567* Δ*lacZ4787*(::rrnB-3) λ^−^ Δ*ruvC789*::*aphA2*(Km^R^) *rph-1* Δ(*rhaD*-*rhaB*)568 *hsdR514*	Δ*ruvA786::kan*	Km^R^	22
JW1852	F^−^ Δ(*araD*-*araB*)*567* Δ*lacZ4787*(::*rrnB*-*3*) λ^−^ Δ*ruvC789*::*aphA2*(Km^R^) *rph-1* Δ(*rhaD*-*rhaB*)*568 hsdR514*	Δ*ruvC789::kan*	Km^R^	22

a UB1637 and UB5201 have a novel *recA* allele encoding RecA L77P, with numbering of the RecA protein sequence starting at alanine 2 (20).

**TABLE 4:** Plasmids used in the study

Plasmid	Description	Resistance phenotype^*a*^	Reference
pRMH977	IS*26* in pUC19	Ap	5
pRMH977-F9A	pRMH977 encoding Tnp26 F9A	Ap	14
pRMH977-F4AF9A	pRMH977 encoding Tnp26 F4AF9A	Ap	14
pRMH977-K23A	pRMH977 encoding Tnp26 K23A	Ap	This study
pRMH977-E30P	pRMH977 encoding Tnp26 E30P	Ap	14
pRMH977-G39I	pRMH977 encoding Tnp26 G39I	Ap	14
pRMH977-G39W	pRMH977 encoding Tnp26 G39W	Ap	14
pRMH977-R49A	pRMH977 encoding Tnp26 R49A	Ap	14
pRMH977-W50A	pRMH977 encoding Tnp26 W50A	Ap	14
pRMH977-W50P	pRMH977 encoding Tnp26 W50P	Ap	14
pRMH977-Y54A	pRMH977 encoding Tnp26 Y54A	Ap	14
pRMH977-S158	pRMH977 codon 158 TCA	Ap	This study
pRMH977-K210A	pRMH977 encoding Tnp26 K210A	Ap	This study
pRMH1013	IS*1006*/*1008* in pUC19	Ap	12
R388::IS*26*	R388 with IS*26*	SuTp	5
R388::IS*26*-F4A	R388::IS*26* encoding Tnp26 F4A	SuTp	14
R388::IS*26*-F9A	R388::IS*26* encoding Tnp26 F9A	SuTp	14
R388::IS*1008*	IS*1008* in R388	SuTp	12

a Abbreviations: Ap, ampicillin; Su, sulfamethoxazole; Tp, trimethoprim.

### Cointegration assays and statistical analyses.

Mating-out assays were conducted to detect the fusion of nonconjugative pUC19 (Ap^R^) derivatives and conjugative R388 (Tp^R^) derivatives in various backgrounds, as described previously (5). The cointegration frequency was calculated as the ratio of transferred cointegrates per transconjugants, e.g., with UB5201(Nx^R^) as donor and UB1637(Sm^R^) as recipient, with Sm^R^ Ap^R^ Tp^R^ cointegrates per Sm^R^ Tp^R^ transconjugants. One-way ANOVA and Šidák multiple-comparison tests were performed using GraphPad Prism version 8.3.0 (GraphPad Software, San Diego, CA, USA). *P* values of <0.05 were considered statistically significant.

### PCR mapping of targeted cointegrates and sequencing.

Single colonies recovered as transconjugants were purified by restreaking for single colonies on plates containing the appropriate selective antibiotics. Plasmid DNA was extracted by alkaline lysis and used as the template in PCR amplification conducted under standard PCR conditions, as previously described (19). Amplification products were visualized after gel electrophoresis with appropriate standards and controls. Targeted cointegrate formation between pRMH977 and R388:IS*26* derivatives was verified by PCR screening using published primer pairs (5) to amplify each IS*26* copy, as previously described. PCR amplicons were sequenced by the Sydney node of the Australian Genome Research Facility Ltd. on an Applied Biosystems 3720*xl* DNA Analyser using the BigDye Terminator system.
